# Measles susceptibility in young Thai men suggests need for young adult measles vaccination: a cross sectional study

**DOI:** 10.1186/s12889-016-2987-z

**Published:** 2016-04-11

**Authors:** Siriphan Gonwong, Thippawan Chuenchitra, Patchariya Khantapura, Dilara Islam, Carl J. Mason

**Affiliations:** Department of Enteric Diseases, Armed Forces Research Institute of Medical Sciences, 315/6 Rajvithi Road, Bangkok, 10400 Thailand; Division of Research, Armed Forces Research Institute of Medical Sciences, Bangkok, 315/6 Rajvithi Road, Bangkok, 10400 Thailand

**Keywords:** Measles, Seroprevalence, Thailand, Young adult, Disease susceptibility, Vaccination

## Abstract

**Background:**

Measles remains a major public health concern in Thailand despite the introduction of vaccination since 1984. Similar to other countries, Thailand has experienced numerous measles outbreaks including adult communities such as university student dormitories, prisons, refugee camps, and military recruit camps. These outbreaks raise questions on the seroprotective antibody level in Thai adults.

**Methods:**

To better understand measles susceptibility in young Thai adults, a retrospective measles seroprevalence study on repository serum specimens obtained with informed consent from young Thai men entering the Royal Thai Army (RTA) during 2007–2008 was conducted. A total of 7760 stratified randomized samples were chosen by residence province. Measles IgG titer was measured using a commercial IgG quantitative ELISA kit following the manufacturer’s instructions. An antibody level ≥ 250 International Units per Liter (IU/L) was interpreted as seropositive.

**Results:**

The overall measles seroprevalence was 78.5 % (95 % Confidence Interval: 77.6–79.4 %) with geometric mean titer of 738 IU/L (95 % Confidence Interval: 716–760 IU/L). The measles seroprevalence by province ranged from 59.6 % to 93.1 %. A trend of decreasing seroprevalence in the younger cohorts despite increasing immunization coverage was found. Lower seroprevalence than vaccination coverage was observed in the youngest age group.

**Conclusions:**

To achieve long term measles control and elimination, an integrated two doses vaccination strategy has been implemented in children in Thailand. This nationwide measles seroprevalence study in young adult RTA recruits found a measles seroprevalence lower than WHO’s recommendation for measles outbreak prevention and elimination. These results raise concerns for measles control in Thailand. Supplementary immunization in young adults is essential especially in high-risk and densely populated communities to establish herd immunity for outbreak prevention and elimination.

## Background

Measles is highly contagious and the cause of mortality and morbidity with an estimated 74,200 and 37,500 deaths reported annually in Africa and South-East Asia, respectively. Though measles vaccine has been available for over 40 years and global deaths reduced from an estimated 2.6 million in 1980 to 145,700 in 2013, measles remains a public health burden especially in developing countries. The deaths are primarily in children who are either unvaccinated or under-vaccinated [1, 2]. However, measles outbreaks have been sporadically reported in developed countries with high vaccination coverage under the two doses vaccine strategy suggesting that communities of susceptible individuals allows measles transmission [1].

In Thailand, measles immunizations were started in 9 month olds in 1984 as part of the Expanded Program on Immunization (EPI) program. One dose measles vaccine coverage escalated from 5 % in 1984 to 52 % in 1987 and has exceeded 90 % since 1995 [3, 4]. A second dose of measles vaccine was added in 1996 for first grade students and replaced by measles-mumps-rubella (MMR) vaccine in 1997. Two dose measles vaccine coverage has increased from 86 % in 2001 to 94 % in 2014 [4]. The number of reported measles cases in the Annual Epidemiology Surveillance Report from the Ministry of Public Health, Thailand, has showed a declined since the start of the vaccination program from 93.67 per 100,000 people in 1984 to 4.10 per 100,000 people in 2013 [3, 5]. The highest incidence reported in 2013 was in the 0–4 years age group (20.70 per 100,000 people), followed with 5–9 years age group (7.73 per 100,000 people) and 15–24 years age groups (6.59 per 100,000 people) [5]. Similar to other countries, Thailand has experienced numerous measles outbreaks long after the introduction of vaccination. Measles outbreaks have been reported in many populations including children and adult populations such as school age children, university students, factory workers, military recruits, medical staff, and prisoners; thereby, questioning the seroprotective antibody levels in the adult Thai population [5–8]. Seroprevalence data in Thailand from endemic diseases including measles is limited; the available studies generally cover small populations [9]. This retrospective study of nationwide measles seroprevalence was conducted on randomized repository serum specimens from Royal Thai Army (RTA) recruits to better understand measles susceptibility in young Thai adults.

## Methods

### Specimen cohort

Approximately 60,000 young Thai men are selected for enlistment in the RTA by lottery at the district level annually. Men exempted from the lottery include the handicapped, certain religious personnel, government teachers and individuals who complete military instruction or alternative military service. The selected men are approximately a 10 % random sample at the district level of all young Thai men [10]. From a total of 121,370 young men entering the RTA during 2007–2008, 98.4 % joined a nationwide HIV-1 surveillance program under informed consent with permission for future studies. This study was approved by the Institutional Review Board, Royal Thai Army Medical Department, Bangkok, Thailand (S066h/52) and approved as exempt by the Human Subjects Protection Branch, Walter Reed Army Institute of Research, Washington, DC, USA (WRAIR#1639). The study was conducted on these 2007–2008 repository serum specimens from young Thai men aged 18 to 30 years, born between 1977 and 1990. The men aged years 24 and younger by calculations would have been the first cohort offered two doses of measles vaccine as part of the EPI in 1984 (first dose) and 1991 (second dose at age of 6), whereas the men aged 25–30 years were born prior to the measles vaccination program initiation and missed the second dose program [3]. Sample sizes were calculated to detect a seroprevalence of 50 % in each of the 76 provinces to within 10 % of the true value with 95 % confidence. A total of 7,760 stratified randomized samples were chosen on the basis of reported residence province prior to the RTA entry.

### Serological test

Measles IgG titer was measured using a commercial IgG quantitative ELISA kit following the manufacturer’s instructions (EUROIMMUN, Luebeck, Germany). The standard curve is obtained by point to point plotting of the extinction value unit measured for the 4 calibrators against the corresponding unit at 50, 250, 1000 and 5000 international unit per liter (IU/L). Study sera and kit internal controls were tested and interpreted according to the manufacturer’s instructions where an antibody level equal to or greater than 250 IU/L was interpreted as seropositive. Individual sera with a value below the cut-off value of 250 IU/L recommended by EUROIMMUN are considered non-infected.

### Statistical analysis

Associations between the available demographic characteristics (age, marital status, education level, residential area and region of residence) and measles seroprevalence were tested (Pearson’s chi square) with two-tailed and *p*-value < 0.05 considered statistically significant, analyses were performed using SPSS version 12 (SPSS Inc; Chicago, Illinois). A spatial distribution map of measles seroprevalence across Thailand by province of residence was generated using ArcView 8.3 (ESRI, Redlands, CA).

## Results

**Fig. 1:**
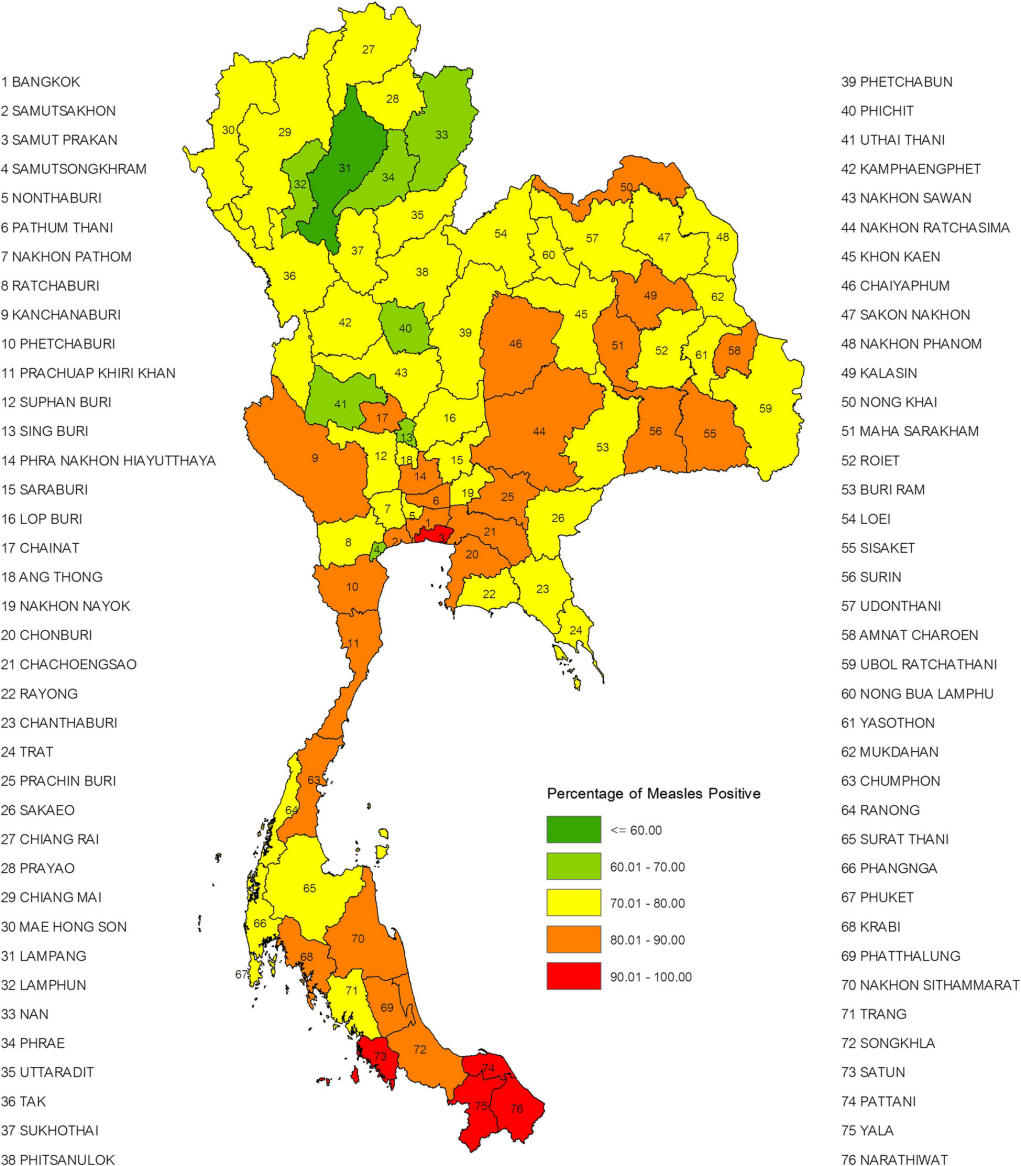
Choropleth map of measles seroprevalence in young Thai men, 2007–2008. Prevalence is stratified by color and location determined by reported residence province during the 2 years prior to Royal Thai Army enlistment. Thailand shapefile in the public domain [25]

**Fig. 2:**
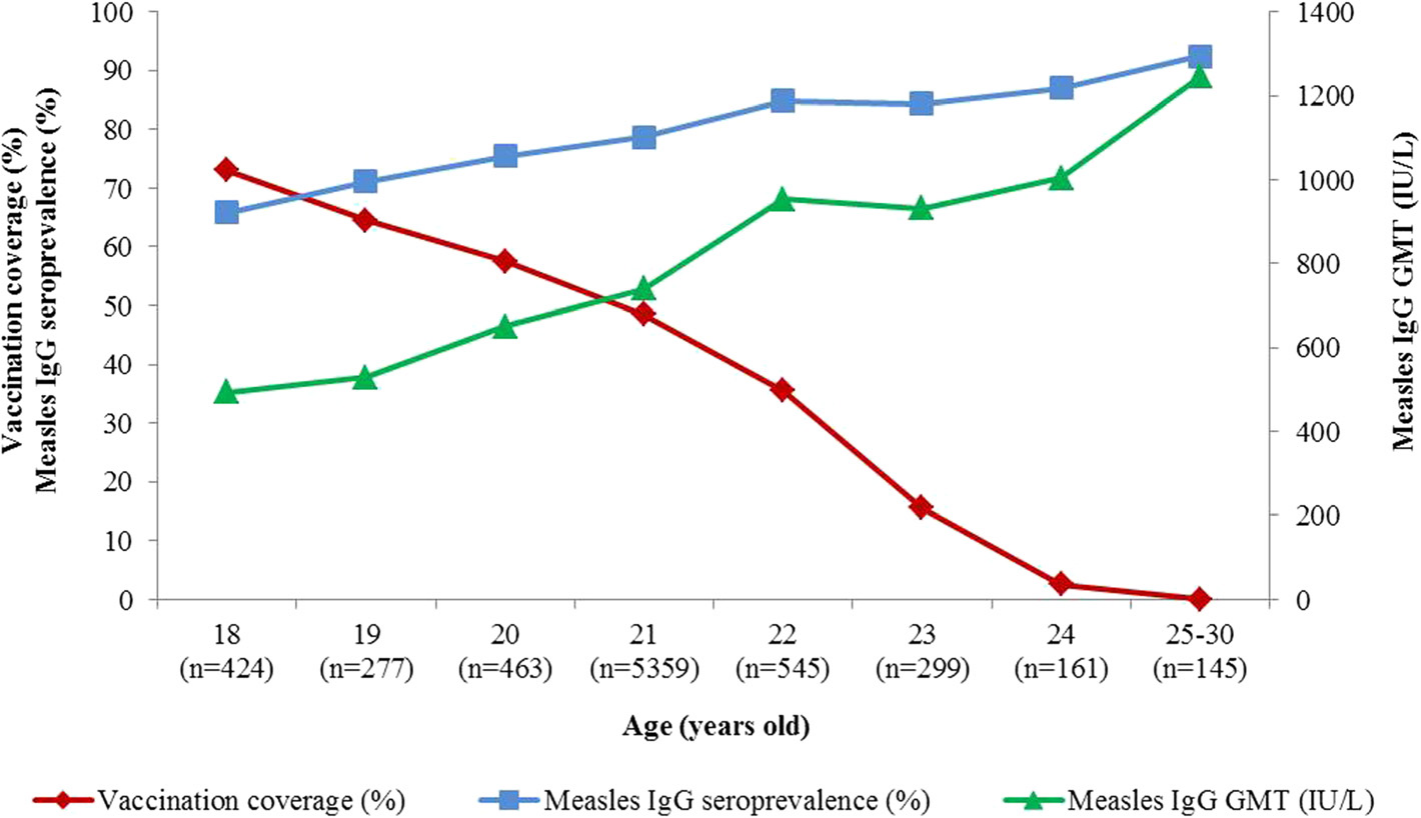
Measles vaccine coverage, measles seroprevalence and geometric mean titer (GMT) in young Thai men, 2007–2008. Vaccination coverage data at 1 year of age from reference 12 by the WHO. Age is reported age in years at time of entry to the Royal Thai Army. Measles seroprevalence defined as percent with titer >250 International Units per Liter (IU/L) by IgG ELISA

The study population is described in the Table 1. The majority of RTA conscripts were 21 years of age, unmarried, junior high school graduates and lived in rural areas. The sample size per province ranged from 69–130. The overall IgG measles seroprevalence was 78.5 % (95 % Confidence Interval (CI): 77.6 %–79.4 %) and geometric mean titer (GMT) was 737.9 IU/L (95 % CI: 716.4–760.1). The measles seroprevalence by residential province ranged from 59.6 % to 93.1 %, 31 of 76 provinces had a seroprevalence ≥ 80 % and only 5 provinces had a seroprevalence ≥ 90 %. Choropleth map of measles seroprevalence by residential province is shown in Fig. 1. The measles seroprevalence was lowest in the Northern region and highest in the Southern region. In the univariate analysis, measles IgG seropositive was significantly associated with age group, education level and residence region, but not marital status or type of residence (Table 1). The measles seroprevalence ranged from 92.4 % with GMT 1245 (95 % CI: 1036–1497) in the oldest age group to 65.8 % with GMT 493 (95 % CI: 437–555) in the youngest age group. Measles vaccine coverage, measles seroprevalence and GMT in young Thai men, 2007–2008, are shown in Fig. 2.

**Table 1:** Measles seroprevalence in association with demographic variables in young Thai men, 2007-2008

Demographic variables	Number Tested^a^	Measles seroprevalence (%) (95 % CI)^b^	GMT^c^ (IU/L)^d^ (95 % CI)
Age group (years)^e^	7673		
18–20	1164	70.9 (68.3–73.5)	560 (520–603)
21	5359	78.7 (77.6–79.8)	740 (714–767)
22–24	1005	85.0 (82.8–87.2)	955 (885–1032)
25–30	145	92.4 (88.1–96.7)	1245 (1036–1498)
Marital status	7576		
Unmarried	6067	78.2 (77.1–79.2)	733 (709–758)
Married	1509	80.4 (78.4–82.4)	776 (727–829)
Education level^e^	7743		
Primary school and less	2121	79.2 (77.4–80.9)	783 (739–830)
Junior high school	2641	77.7 (76.1–79.3)	706 (672–742)
High school to diploma	2676	77.8 (76.2–79.3)	712 (677–749)
Bachelor	305	86.2 (82.4–90.1)	962 (838–1106)
Residential area	6399		
Rural	3896	78.3 (77.0–79.6)	745 (707–785)
Municipal (Urban)	2503	79.3 (77.8–80.9)	739 (709–770)
Region of residence^e^	7760		
North	1738	71.7 (69.6–73.8)	575 (539–613)
Central	2626	79.0 (77.5–80.6)	732 (696–770)
Northeast	2004	79.5 (77.8–81.3)	771 (728–817)
South	1392	84.3 (82.4–86.2)	961 (899–1027)

^a^Number in each demographic characteristic does not add to the total number of study samples because of missing data; ^b^CI, confidence interval; ^c^ GMT, geometric mean titer; ^d^IU/L, international unit per liter; ^e^ χ^2^ test statistically significant (Pearson’s chi square, two-sided, *p* <0.05)

## Discussion

This study represents a nationwide seroprevalence study of measles IgG antibodies in young Thai men. The overall, by province, and by age cohort measles seroprevalence in these young men was below the 93–95 % recommended by the WHO for population immunity [11]. These findings may explain partially the measles outbreaks occurring in adult populations in Thailand. Our study is consistent with previous measles seroprevalence studies conducted in Thailand. A study of a Thai population sample from the same birth years (1988–1992) had a similar measles seroprevalence [9].

We found significant difference of seroprevalence and GMT amongst the four regions of Thailand where the South exhibited the highest and the North the lowest. Although the geography of the Northern region of Thailand is mountainous with population scattered amongst hilly areas [12] whereas the Southern region is a peninsula between seas with population concentrated in the coastal areas, with limited regional vaccine coverage data in the early years of measles immunization as well as inconsistent case reporting by region; we cannot determine why measles seroprevalence is highest in the Southern region [5, 6, 13].

This study population consists of young Thai men born as the measles vaccination program was initiated in Thailand. The measles vaccine coverage ranged from 0 % in the oldest age group born before measles vaccine program initiation to a reported coverage of 73.0 % in the youngest age group in Thailand (Fig. 2). The higher seroprevalence and GMT were found in the oldest age group was the result of natural infection (born before vaccination), while the successively lower seroprevalence and GMT in the younger age groups was from a combination of vaccine induced immunity and natural infection. However, these observations are limited by lack of individual measles vaccination and infection history in this population.

Low seroprevalence of measles antibodies may be from inadequate vaccine coverage, vaccination failure, or waning immunity. Inadequate vaccine coverage may be caused by several factors such as lack of vaccine access, missed vaccination opportunities, and ignorance of the importance of vaccination. Vaccination failures may be attributable to the immaturity of the immune system, residual maternal antibodies, inadequate vaccine dose, or vaccine inefficacy [14]. The seroprotective level increases from 85 % in children given first dose vaccine at 9 months to 95 % at 12 months. According to the EPI in Thailand, children were vaccinated with the first dose of the measles vaccine at the age of 9 months as recommended by WHO for countries with ongoing measles transmission; previous serological studies in Thailand showed that the passive maternal antibody was rapidly lost in infants starting from 4–6 months and almost completely absent at 8–9 months [15]. A second later dose may cover the primary non-responders and unvaccinated persons who would otherwise accumulate over time and allow transmission [1]. This may explain why Thais are still susceptible to measles infection with 40 % of reported cases found in children of pre-school age (2009–2013). Recently, the Ministry of Public Health, Thailand announced that from 2015 onwards the 2nd dose of vaccination previously given to first graders (6 years of age) will be given to children at 2.5 years of age instead [16]. The waning of protective immunity could pose a risk in communities receiving a single dose of immunization at a young age without ongoing natural exposure [17]. A lower measles seroprevalence than vaccination coverage found in this study may reflect a combination of waning immunity and measles vaccine failure in this Thai population. Usually waning immunity is age related in populations without natural infection or supplemental vaccinations, but this is not seen in our study cohort [18].

In Thailand, immunizations are routinely administered to children regardless of gender and no supplementary measles immunizations were given to the recruits upon entering the RTA; this nationwide measles seroprevalence study in a sample of Thai recruits selected by lottery at the district level may represent the measles protective immunity levels in the general young adult population of the same age groups [3]. Our results show that approximately 20 % of Thai recruits in 2007–2008 are susceptible to measles; we speculate that females born in the same years may present similar trends in measles seroprevalence raising concerns for young Thai women of reproductive age even through a slightly higher measles seroprevalence in females has been reported in studies elsewhere [19–21].

With low herd immunity, measles infection can spread easily in densely populated communities such as prisons, dormitories, recruit camps and schools. WHO’s recommendation for immunization coverage sufficient to reduce measles mortality is ≥90 % at national level and ≥80 % at every district level. For elimination, each district must achieve vaccine coverage ≥95 % through a two-dose vaccination program [11]. In 2001, a measles outbreak in Bangkok amongst healthcare workers emphasized the ease of transmission within high-risk adult populations [7]. A supplementary immunization activity for higher risk adult communities may be an effective preventive measure, as implemented in Norway. Even with the two doses of MMR vaccine at the ages of 15 months and 12–13 years as part of Norway’s national childhood immunization program where vaccine coverage was higher than 95 %, sporadic incidence of measles was still reported [22, 23]. An additional dose of mump-measles-rubella vaccine has also been incorporated into the required vaccination program for military conscripts in Norway as an outbreak prevention measure [23].

Limitations of this study include a single sample collection from 2007–2008 in a population of all male Thai recruits. The lack of information on individual childhood measles as well as measles vaccination history and the measles vaccine strains limits the conclusions of this study. For future studies, it will be important to compare seroprevalence data on samples collected more recently from individuals with documented immunization histories from several age groups in the general population to better represent current Thai population protective immunity.

## Conclusion

This nationwide measles seroprevalence study from RTA recruits born between 1977 and 1990, found a measles seroprevalence of 78.5 %, lower than WHO’s recommendation for measles outbreak prevention and elimination. A trend of decreasing seroprevalence in the younger cohorts in contrast to increasing immunization coverage was found. These results raise concerns for measles control in Thailand as well as meeting the Southeast Asia plan for measles control and elimination goal by 2020 [24]. While the two doses immunization strategy in childhood has been implemented, a supplementary immunization activity in young adults is essential especially in high-risk and densely populated communities to establish herd immunity for outbreak prevention and ultimately measles elimination in Thailand.
